# Association between dietary niacin intake and chronic obstructive pulmonary disease among American middle-aged and older individuals: A cross-section study

**DOI:** 10.1371/journal.pone.0312838

**Published:** 2024-11-21

**Authors:** Yushan Shi, Shuangshuang Pu, Chunlai Zhang, Kanghong Xu, Xuxiao Guo, Wei Gao

**Affiliations:** Department of Laboratory Medicine, Affiliated Hospital of Shandong University of Traditional Chinese Medicine, Shandong, China; School of Pharmacy, Ardabil University of Medical Sciences, ISLAMIC REPUBLIC OF IRAN

## Abstract

**Background:**

The attention towards the relationship between chronic obstructive pulmonary disease (COPD) and dietary intake is escalating. However, the effects of dietary niacin on COPD in middle and older individuals remains unclear. This study aimed to illuminate the connection between dietary niacin intake and COPD.

**Methods:**

This cross-sectional study analyzed 7,170 participants from the National Health and Nutrition Examination Survey (NHANES) spanning the years 2013 to 2018. Participants were categorized into four groups based on quartiles of dietary niacin intake. To examine the association between covariates, dietary niacin intake, and COPD, we employed univariate analysis and multivariate logistic regression equations. Additionally, restricted cubic splines were utilized to assess linearity. Furthermore, we conducted stratified and interaction analyses to evaluate the stability of the relationship in diverse subgroups.

**Results:**

Among the 7,170 participants, 11.6% (834/7170) were diagnosed with COPD. The multivariable adjusted odds ratios (ORs) and 95% confidence intervals (CIs) for COPD were 0.96 (95% CI: 0.77–1.19, p = 0.706), 0.78 (95% CI: 0.62–0.99, p = 0.038), and 0.76 (95% CI: 0.57–1.00, p = 0.047), respectively, when comparing the second, third, and fourth quartiles of niacin intake levels to the lowest quartile (p for trend = 0.017). An inverse association was observed between the occurrence of COPD and dietary niacin intake (nonlinear: p = 0.347). Stratified analyses revealed no significant differences or interactions.

**Conclusion:**

Our findings suggest a potential link between increased dietary niacin intake and a decreased prevalence of COPD.

## Introduction

Chronic obstructive pulmonary disease (COPD) encompasses a group of lung disorders marked by enduring respiratory symptoms and airflow limitation [1]. In the United States (US), COPD stands as the leading cause of disability and the third leading cause of death, imposing a substantial burden on clinical and healthcare resources [2, 3]. The anticipated increase in the burden of COPD in the coming decades, attributed to chronic exposure to risk factors and demographic shifts [4, 5]. As such, exploring avenues for the prevention and management of COPD is paramount. Beyond the established preventive measures like cessation of smoking and minimizing exposure to smoke, dietary factors are increasingly acknowledged for their potential influence in the onset and progression of chronic ailments such as COPD [6]. A diet high in vegetables, fruit, and fish is associated with a lower prevalence of COPD, while coffee, and vitamin D intake deficiency is associated with a higher prevalence of COPD [7–9]. As observed in various studies, vitamin supplementation could play a significant role in the management of COPD [10–13].

Niacin, or vitamin B3, is a precursor to nicotinamide adenine dinucleotide (NAD) and nicotinamide adenine dinucleotide phosphate (NADP), which are integral to cellular redox reactions and energy metabolism [14, 15]. Niacin supplementation has demonstrated benefits in experimental models related to cancer, cardiovascular disease, mental health, and oxidant lung injury [14] which are relevant to the systemic manifestations of COPD. Niacin’s role in inflammation and oxidative stress pathways is well-documented, and these pathways are known to be dysregulated in COPD [16]. A recent observational study investigated the relationship between dietary niacin intake and the COPD in the adult population of the United States [17]. Nonetheless, the specific link between niacin intake and COPD prevalence in people older than 40 years has not been extensively explored.

Therefore, the primary aim of this cross-sectional study was to investigate the association between dietary niacin intake and the prevalence of COPD among American middle-aged and older individuals. This study may provide insights for future COPD prevention and treatment strategies.

## Materials and methods

### Data sources

This cross-sectional study utilized data from the National Health and Nutrition Examination Survey (NHANES) spanning the years 2013 to 2018. NHANES is a de-identified and publicly available dataset, participants are selected through a stratified, multistage, probability sampling design. The survey involves home visits, screenings, and laboratory testing conducted by a mobile examination center (MEC). Detailed data can publicly accessible at https://www.cdc.gov/nchs/nhanes/index.htm.

Approval for all procedures in the survey was granted by the National Center for Health Statistics (NCHS) Research Ethics Review Board (Continuation of Protocol #201117), and written informed consent was obtained from all participants. Throughout data collection, we had no access to information that could identify individual participants. Additional Institutional Review Board approval was not required for the secondary analysis. Thus, The Ethics Committee of the Affiliated Hospital of Shandong University of Traditional Chinese Medicine agreed to strike the ethics of this study (No:2024–0006).

### Study participants

**Fig 1:**
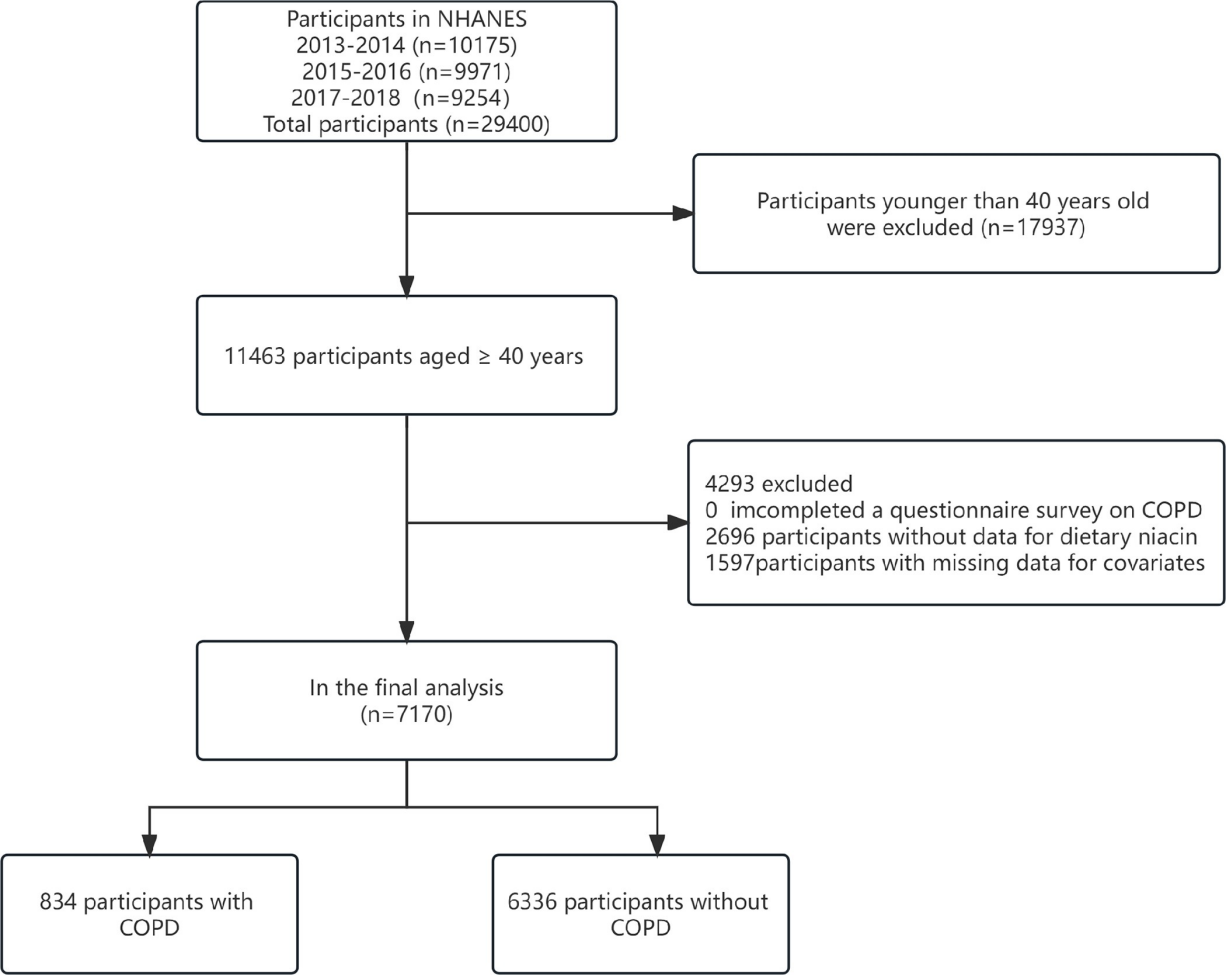
Flow diagram of study participants. Abbreviations: NHANES, National Health and Nutrition Examination Survey; COPD, chronic obstructive pulmonary disease.

In this study, we used publicly available NHANES data generated from surveys conducted in 2013–2014, 2015–2016, and 2017–2018, in which a total of 29,400 people provided demographic data. We excluded individuals age < 40 years (n = 17937), missing data on COPD (n = 0), missing data on dietary niacin intake (n = 2696), we further excluded individuals missing data on covariates(n = 1597), which including family income (n = 831), body mass index (n = 87), education level (n = 4), marital status (n = 1), serum cotinine (n = 289), results of the questionnaire in hypertension (n = 8), high cholesterol (n = 63), diabetes (n = 280), coronary heart disease (n = 25), stroke (n = 6), cancer (n = 3). Ultimately, 7170 participants were included in the further analyses. The detailed inclusion and exclusion process is shown in **Fig 1.**

### Measurement of dietary niacin intake

The dietary interview component, known as What We Eat in America (WWEIA), is a collaborative effort between the US Department of Agriculture (USDA) and the US Department of Health and Human Services (DHHS). It involves administering two 24-hour dietary recall interviews. The first dietary recall is conducted face-to-face at the Mobile Examination Center (MEC), while the second recall is carried out via a telephone interview approximately 3 to 10 days after the initial recall.

To calculate the nutrients and food components in all reported food items, the USDA’s Food and Nutrient Database for Dietary Studies (FNDDS) was employed. The dataset on total nutrient intakes provided a summary record of nutrient intake for each individual. Nutrients obtained from dietary supplements or medications were excluded from the nutrient estimates. This approach ensured a comprehensive and accurate assessment of dietary niacin intake from natural food sources without the confounding influence of supplemental sources. In this study, the daily niacin intake of participants was determined by calculating the average of their two dietary recalls.

### COPD outcomes

The COPD outcome in this study was defined as a composite of three self-reported COPD conditions: emphysema, chronic bronchitis, and COPD itself [18–20]. A participant was categorized as having COPD if they responded "yes" to any of the three questions during the standardized medical condition questionnaire administered in the personal interview. The questions included, "Have you ever been told that you have emphysema/chronic bronchitis/chronic obstructive pulmonary disease?"

### Assessment of covariates

Various potential covariates were included based on the published literatures [21–23]. We included demographic data, lifestyle, body measurements, chronic diseases, and laboratory examinations. The present study included the following demographic covariates: age, sex (female, male), race (Mexican American, non-Hispanic white, non-Hispanic black, others), education level (<high school, high school, >high school) [24], marital status (married, living with a partner, living alone), and family income. The poverty income ratio (PIR) categorized family income into three groups: low (PIR ≤ 1.3), medium (1.3 < PIR ≤ 3.5), and high (PIR > 3.5) [25]. In lifestyle, smoking status were categorized as never smoked (smoked less than 100 cigarettes in their lives), former smoker (quit smoking after smoking more than 100 cigarettes), or current smoker [26]. Recreational physical activities were categorized as sedentary, moderate, vigorous (vigorous recreational activities were defined as yes to the question: do you do any vigorous-intensity sports, fitness, or recreational activities that cause a small increase in breathing or heart rate) [22]. Body measurements included body mass index (BMI), which was calculated by dividing weight (kg) by height in square meters, and it was categorized as less than 25, 25 to 30, and more than 30kg/m^**2**^ [27]. Chronic diseases include hypertension, high cholesterol diabetes, stroke, coronary heart disease, and cancer, defined as yes or no. Information regarding total energy intake was acquired through the 24-hour dietary recall. laboratory examinations included serum cotinine(ng/mL).

### Statistical analysis

Categorical variables were presented as absolute values (n) or percentages (%), while continuous variables were described using the mean ± standard deviation (mean ± SD) or median (interquartile range, IQR). Group differences were assessed through one-way analyses of variance for normal distribution, Kruskal–Wallis tests for skewed distribution, and chi-square tests for categorical variables.

Univariate logistic regression analyses were employed to explore risk factors for COPD prevalence. Multivariate logistic regression models were utilized to determine odds ratios (OR) and 95 percent confidence intervals (95% CIs) regarding the relationship between dietary niacin intake and COPD. The crude model was unadjusted. Model 1 incorporated adjustments for age, sex, and race/ethnicity. Model 2 further adjusted for family income, physical activity, smoking status, education level, marital status, body mass index, serum cotinine, and total energy. Model 3 represented a fully adjusted model, including sex, age, race/ethnicity, family income, physical activity, smoking status, education level, marital status, body mass index, serum cotinine, total energy, hypertension, high cholesterol, diabetes, coronary heart disease, stroke, and cancer.

Restricted cubic spline analysis examined nonlinear associations between dietary niacin intake and COPD, adjusting for all potential confounding variables in model 3, with four knots positioned at the 5th, 35th, 65th, and 95th percentiles.

Interaction and subgroup analyses were conducted to explore potential confounding factors influencing the association between niacin intake and COPD. Subgroup analyses were stratified by sex (female or male), age (<65 or ≥65 years), smoking status (never, former, current), hypertension (yes or no), and diabetes (yes or no).

To assess the robustness of the results, we performed several sensitivity analyses. To assess the impact of the outlier on our findings, we excluded participants with extreme energy intake (<500 or >5000 kcal per day) to mitigate the influence of potential outliers(25, 27). We also excluded individuals whose niacin intake exceeded the sample population mean ± 3 standard deviations (SD).

Furthermore, we employed multiple imputation (MI) methods by chained equations method to address the missing data, followed by a multifactor regression analysis of the imputed dataset. Finally, given that the NHANES database utilizes a complex multi-stage sampling methodology, we performed a weighted regression analysis to test the robustness of our main findings. The sampling weights for 2013–2018 were calculated as follows: 1/3 × WTDR2D.

All statistical analyses were performed using R software (version 4.2.2) and Free Statistics software version 1.9 [28]. Two-sided p < 0.05 was considered for statistical significance.

## Results

### Baseline characteristics of participants

The baseline characteristics of 7,170 subjects according to their niacin intake quartiles were shown in **Table 1.** The COPD adults accounted for 11.6% (834/7170). The mean age of the population in this study was 59.5 ± 11.5 years, males constituted 47.5% of the study population, and 43.3% were White. Higher niacin intake is likely to be younger age, male gender, marital status of being married, never smokers, have higher education, have higher body mass index. Additionally, these individuals were less likely to report histories of coronary heart disease, stroke, hypertension, diabetes, or cancer. The baseline characteristics of participants were also summarized in **S1 Table** according to whether they have COPD. Members in COPD group were more likely older (62.9 ± 11.5 years vs 59.1 ± 11.9 years, *p*< 0.001), and had a lower average daily niacin intake (22.2 ± 11.2mg/d vs 24.0 ± 11.7 mg/d, *p*< 0.001).

**Table 1:** Population characteristics by quartiles of dietary niacin intake.

Characteristic	Total (n = 7170)	Niacin Intake, mg/d				*p*
		Q1(≤16.08) (n = 1793)	Q2(16.09–21.84) (n = 1792)	Q3(21.85–28.97) (n = 1792)	Q4 (≥28.98) (n = 1793)	
Sex, n (%)						< 0.001
Male	3407 (47.5)	487 (27.2)	687 (38.3)	917 (51.2)	1316 (73.4)	
Female	3763 (52.5)	1306 (72.8)	1105 (61.7)	875 (48.8)	477 (26.6)	
Age, years, Mean ± SD	59.5 ± 11.9	61.4 ± 11.8	60.5 ± 12.1	59.5 ± 12.0	56.6 ± 11.2	< 0.001
Physical activity, n (%)						< 0.001
Sedentary	3900 (54.4)	1067 (59.5)	978 (54.6)	936 (52.2)	919 (51.3)	
Moderate	2048 (28.6)	516 (28.8)	522 (29.1)	549 (30.6)	461 (25.7)	
Vigorous	1222 (17.0)	210 (11.7)	292 (16.3)	307 (17.1)	413 (23.0)	
Smoking status, n (%)						< 0.001
Never	3817 (53.2)	992 (55.3)	1012 (56.5)	953 (53.2)	860 (48.0)	
Former	2145 (29.9)	492 (27.4)	515 (28.7)	539 (30.1)	599 (33.4)	
Current	1208 (16.8)	309 (17.2)	265 (14.8)	300 (16.7)	334 (18.6)	
Race/ethnicity, n (%)						< 0.001
Mexican American	926 (12.9)	231 (12.9)	234 (13.1)	222 (12.4)	239 (13.3)	
Non-Hispanic white	3106 (43.3)	700 (39.0)	787 (43.9)	807 (45.0)	812 (45.3)	
Non-Hispanic black	1501 (20.9)	444 (24.8)	380 (21.2)	349 (19.5)	328 (18.3)	
Others	1637 (22.8)	418 (23.3)	391 (21.8)	414 (23.1)	414 (23.1)	
Education level, n (%)						< 0.001
Less than high school	1402 (19.6)	443 (24.7)	353 (19.7)	314 (17.5)	292 (16.3)	
High school	1670 (23.3)	435 (24.3)	413 (23.0)	430 (24.0)	392 (21.9)	
More than high school	4098 (57.2)	915 (51)	1026 (57.3)	1048 (58.5)	1109 (61.9)	
Marital status, n (%)						< 0.001
Married	4211 (58.7)	949 (52.9)	1036 (57.8)	1110 (61.9)	1116 (62.2)	
living with a partner	331 (4.6)	68 (3.8)	82 (4.6)	76 (4.2)	105 (5.9)	
Living alone	2628 (36.7)	776 (43.3)	674 (37.6)	606 (33.8)	572 (31.9)	
Family income, n (%)						< 0.001
Low (PIR<1.3)	1970 (27.5)	600 (33.5)	464 (25.9)	470 (26.2)	436 (24.3)	
Medium (1.3≤PIR<3.5)	2802 (39.1)	714 (39.8)	707 (39.5)	696 (38.8)	685 (38.2)	
High (PIR≥3.5)	2398 (33.4)	479 (26.7)	621 (34.7)	626 (34.9)	672 (37.5)	
Body mass index, n (%)						0.299
<25 kg/m2	1694 (23.6)	406 (22.6)	453 (25.3)	408 (22.8)	427 (23.8)	
25 to <30 kg/m2	2432 (33.9)	594 (33.1)	593 (33.1)	617 (34.4)	628 (35)	
≥30 kg/m2	3044 (42.5)	793 (44.2)	746 (41.6)	767 (42.8)	738 (41.2)	
Total energy, Kcal, Mean ± SD	2028.3 ± 921.1	1352.2 ± 549.2	1813.0 ± 627.3	2142.0 ± 671.5	2806.0 ± 1061.6	< 0.001
Serum cotinine, ng/mL, Median (IQR)	0.03 (0.01, 0.51)	0.03(0.01,0.56)	0.02 (0.01, 0.29)	0.02 (0.01, 0.30)	0.03 (0.01,1.97)	< 0.001
Hypertension, n (%)	3465 (48.3)	926 (51.6)	883 (49.3)	876 (48.9)	780 (43.5)	< 0.001
High cholesterol, n (%)	3391 (47.3)	840 (46.8)	849 (47.4)	884 (49.3)	818 (45.6)	0.162
Diabetes, n (%)	1470 (20.5)	420 (23.4)	346 (19.3)	365 (20.4)	339 (18.9)	0.003
Coronary heart disease, n (%)	466 (6.5)	126 (7)	122 (6.8)	111 (6.2)	107 (6)	0.53
Stroke, n (%)	389 (5.4)	134 (7.5)	92 (5.1)	87 (4.9)	76 (4.2)	< 0.001
Cancer, n (%)	1013 (14.1)	258 (14.4)	291 (16.2)	238 (13.3)	226 (12.6)	0.011
Niacin Intake, mg/d, Mean ± SD	23.8 ± 11.6	12.1 ± 2.9	19.0 ± 1.6	25.2 ± 2.0	38.9 ± 11.6	< 0.001
COPD, n (%)	834 (11.6)	254 (14.2)	221 (12.3)	189 (10.5)	170 (9.5)	< 0.001

**Abbreviations:** COPD, chronic obstructive pulmonary disease; PIR, family poverty income ratio; Q1–Q4, quartiles based on dietary niacin intake.

### Univariate analyses

**Table 2** displays the results of the univariate analysis, indicating significant associations between COPD and various factors including age, gender, smoking habits, race/ethnicity, marital status, economic status, level of physical activity, and history of hypertension, diabetes, stroke, and cancer.

**Table 2:** Association of covariates and COPD risk.

Variable	OR_95CI	*p*-Value
Sex, n (%)		
Male	1 (reference)	
Female	1.23 (1.07~1.43)	0.005
Age, years	1.03 (1.02~1.03)	<0.001
Physical activity, n (%)		
Sedentary	1 (reference)	
Moderate	0.6 (0.5~0.71)	<0.001
Vigorous	0.31 (0.23~0.4)	<0.001
Smoking status, n (%)		
Never	1 (reference)	
Former	2.81 (2.35~3.36)	<0.001
Current	4.71 (3.89~5.68)	<0.001
Race/ethnicity, n (%)		
Mexican American	1 (reference)	
Non-Hispanic white	3.35 (2.49~4.52)	<0.001
Non-Hispanic black	1.81 (1.3~2.51)	<0.001
Others	1.5 (1.08~2.1)	0.016
Education level, n (%)		
Less than high school	1 (reference)	
High school	1.02 (0.83~1.25)	0.837
More than high school	0.66 (0.55~0.79)	<0.001
Marital status, n (%)		
Married	1 (reference)	
living with a partner	1.32 (0.93~1.87)	0.12
Living alone	1.82 (1.57~2.11)	<0.001
Family income, n (%)		
Low (PIR<1.3)	1 (reference)	
Medium (1.3≤PIR<3.5)	0.61 (0.52~0.72)	<0.001
High (PIR≥3.5)	0.31 (0.25~0.38)	<0.001
Body mass index, n (%)		
<25 kg/m2	1 (reference)	
25 to <30 kg/m2	0.83 (0.68~1.02)	0.083
≥30 kg/m2	1.4 (1.17~1.69)	<0.001
Total energy, kcal,	1 (1~1)	0.041
Serum cotinine	1 (1~1)	<0.001
Niacin Intake, mg/d	0.99 (0.98~0.99)	<0.001
Hypertension (Yes vs No)	1.73 (1.49~2.01)	<0.001
High cholesterol (Yes vs No)	1.5 (1.3~1.74)	<0.001
Diabetes (Yes vs No)	1.72 (1.47~2.03)	<0.001
Coronary heart disease (Yes vs No)	3.94 (3.19~4.87)	<0.001
Stroke (Yes vs No)	2.29 (1.78~2.94)	<0.001
Cancer (Yes vs No)	1.83 (1.53~2.2)	<0.001

Abbreviations: COPD, chronic obstructive pulmonary disease; PIR, family poverty income ratio; Ref: reference.

### Associations between dietary niacin intake and COPD

We conducted four logistical regression models (crude model, model 1–3) to explore the association between dietary niacin intake and COPD. As presented in **Table 3**. In comparison to individuals with lower dietary niacin intake in Q1 (≤16.08 mg/day), the adjusted odds ratios (OR) for COPD in Q2 (16.09–21.84 mg/day), Q3 (21.85–28.97 mg/day), and Q4 (≥28.98 mg/day) were 0.96 (95% CI: 0.77–1.19, p = 0.706), 0.78 (95% CI: 0.62–0.99, p = 0.038), and 0.76 (95% CI: 0.57–1.00, p = 0.047), respectively.

**Table 3:** Multivariate logistic regression analysis of dietary niacin for risk of COPD.

Variable	n. event (%)	Crude model		Model 1		Model 2		Model 3	
		OR (95%CI)	*p*	OR (95%CI)	*p*	OR (95%CI)	*p*	OR (95%CI)	*p*
Quartile									
Q1 (≤16.08)	254 (14.2)	1(Ref)		1(Ref)		1(Ref)		1(Ref)	
Q2(16.09–21.84)	221 (12.3)	0.85 (0.7~1.03)	0.106	0.85 (0.7~1.03)	0.102	0.97 (0.78~1.19)	0.742	0.96 (0.77~1.19)	0.706
Q3(21.85–28.97)	189 (10.5)	0.71(0.58~0.8)	0.001	0.73 (0.59~0.9)	0.003	0.79 (0.63~0.99)	0.041	0.78 (0.62~0.99)	0.038
Q4 (≥28.98)	170 (9.5)	0.63(0.52~0.7)	<0.001	0.72 (0.57~0.9)	0.004	0.76 (0.58~1.00)	0.05	0.76 (0.57~1.00)	0.047
*P* for trend			<0.001		0.001		0.018		0.017

Abbreviations: COPD, chronic obstructive pulmonary disease; Q, quartiles; OR, odds ratio; CI, confidence interval; Ref: reference.

The crude model was not adjusted for covariates.

Model I was adjusted for sex, age, race/ethnicity.

Model2 was adjusted for sex, age, race/ethnicity, family income, physical activity, smoking status, education level, marital status, body mass index, serum cotinine, total energy.

Model 3 was adjusted for sex, age, race/ethnicity, family income, physical activity, smoking status, education level, marital status, body mass index, serum cotinine, total energy, hypertension, high cholesterol, diabetes, coronary heart disease, stroke, cancer.

**Fig 2:**
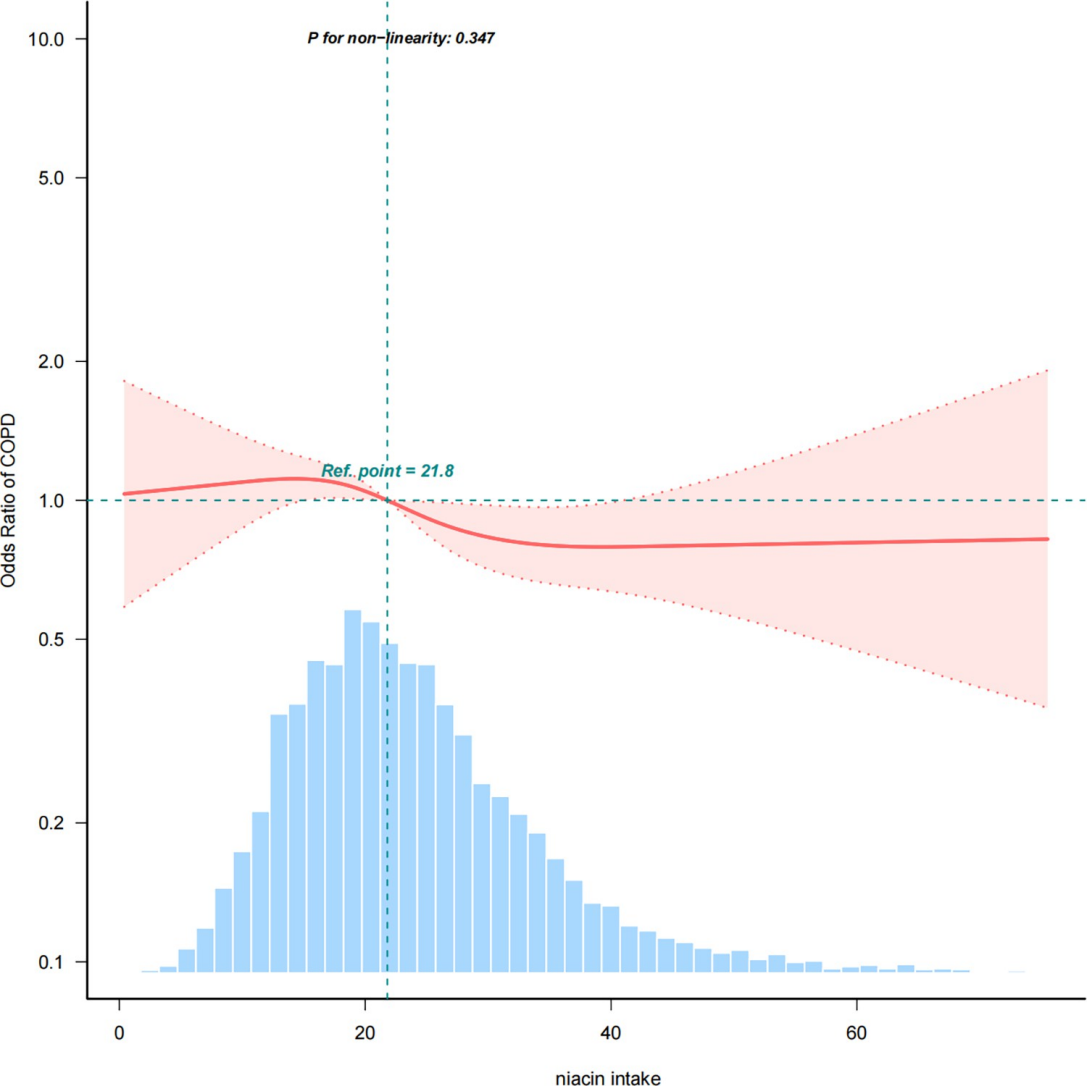
Association between dietary niacin intake and COPD odds ratio. Solid and dashed lines represent the predicted value and 95% confidence intervals. They were adjusted for sex, age, race/ethnicity, family income, physical activity, smoking status, education level, marital status, body mass index, Serum cotinine, total energy, hypertension, high cholesterol, diabetes, coronary heart disease, stroke, cancer. only 99.5% of the data is shown.

The restricted cubic spline analysis illustrated a negative association between dietary niacin intake and COPD incidence, considering all potential confounders (non-linearity: p = 0.347). **Fig 2** provides a visual representation of the association between dietary niacin intake and COPD.

### Subgroup analysis

**Fig 3:**
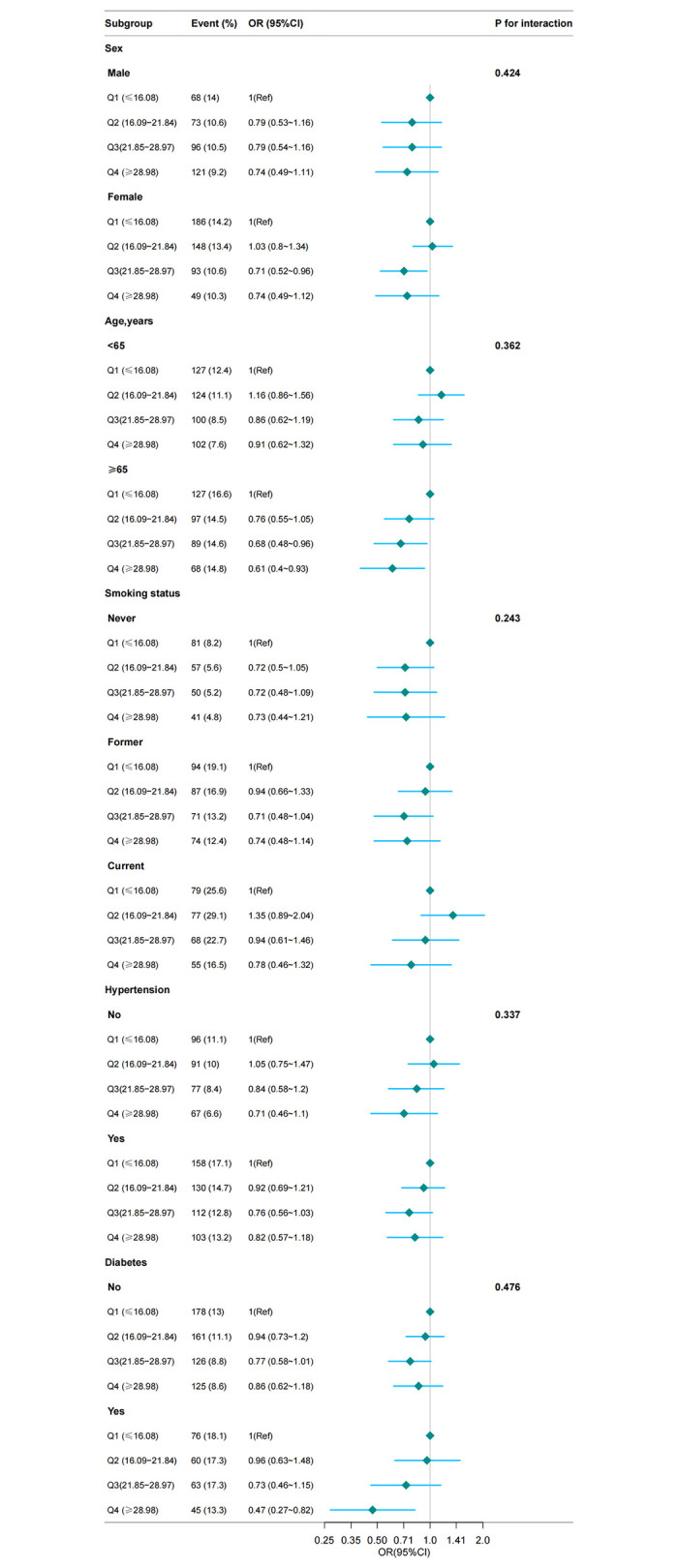
The relationship between dietary niacin intake and COPD stratified using different characteristics. Except for the stratification component itself, each stratification factor was adjusted for all other variables (sex, age, race/ethnicity, family income, physical activity, smoking status, education level, marital status, body mass index, Serum cotinine, total energy, hypertension, high cholesterol, diabetes, coronary heart disease, stroke, cancer.).Abbreviations: COPD, chronic obstructive pulmonary disease; OR, odds ratio; 95% CI, 95% confidence interval.

In this study, we performed analyses on subgroups and interactions to determine the consistency of the association between dietary niacin intake and the prevalence of COPD. As depicted in **Fig 3,** the stratification by age, sex, smoking status, hypertension, and diabetes did not reveal significant interactions, underscoring a stable inverse relationship between niacin intake and COPD risk across different subgroups (P for interaction > 0.05).

### Sensitivity analysis

Firstly, we excluded participants with extreme energy intake (<500 or >5000 kcal per day), the study cohort comprised 7,040 individuals, and the association between dietary niacin intake and COPD remained consistent. In comparison to individuals with lower niacin consumption in Q1 (≤16.14 mg/day), the adjusted odds ratios (OR) for COPD in Q3 (21.84–28.82 mg/day) and Q4 (≥28.83 mg/day) were 0.77 (95% CI: 0.61–0.98, p = 0.033) and 0.75 (95% CI: 0.57–0.99, p = 0.043) (**S2 Table)**, respectively. We also excluded individuals whose niacin intake exceeded the sample population mean ± 3 standard deviations (SD) (**S3 Table),** the association between dietary niacin intake and COPD in 7057 individuals remained consistent.

Further validation through multiple imputation for missing covariates followed by multifactor regression analysis confirmed the robustness of these findings. This analysis, illustrated in **S4 Table**, maintained the inverse relationship between higher dietary niacin levels and decreased COPD prevalence.

As shown in **S5 Table**, the results of the weighted analyses were similar to the unweighted analyses, which confirmed the stability of the inverse association between dietary niacin intake and COPD, even after accounting for the complex sampling design.

## Discussion

The present cross-sectional study, encompassing an analysis of 7,170 participants, has delineated an inverse association between dietary niacin intake and the prevalence of COPD, particularly among American middle-aged and older adults. The subgroup analyses and sensitivity analyses further underscored the robustness of this association. The insights from our study suggest that increasing dietary niacin may be a valuable strategy for families looking to manage COPD risk among their members.

Emerging evidence increasingly emphasizes the impact of diet on obstructive pulmonary disease [5, 29]. Adopting a diet abundant in vitamins, omega-3 fatty acids, fiber, fruits, and vegetables may contribute to a potential reduction in COPD prevalence [30–32]. Despite this, studies exploring the effects of dietary niacin intake on COPD remain scarce. Our study contributes to this evidence base by specifically highlighting the modulatory role of dietary niacin, a B vitamin with established functions in lipid regulation, anti-inflammatory, and antioxidative activities. The observed lower risk of COPD in individuals with higher niacin intake (OR: 0.76; 95% CI: 0.57~1) after adjustment for confounders provides preliminary support for the potential of dietary niacin in COPD prevention and management.

Our study had similar results to a recently published observational study [17], the difference is that previous study population was US adults and we were middle-aged and older adults over the age of 40 years; in addition, previous studies of niacin intake used only one 24-hour recall data, which may have had some effect on the stability of the results, whereas we averaged over the collection of two 24-hour recalls to minimize memory bias. In addition, we adjusted for more confounding and performed some sensitivity analyses to verify the stability of the results. Future prospective studies are warranted to delve deeper into the impact of dietary niacin intake on COPD.

Niacin, a member of the B vitamins group and a precursor of nicotinamide adenine dinucleotide (NAD+), has gained recognition for its use as a lipid-regulating medication to mitigate atherosclerosis [33]. Furthermore, multiple studies have delved into its anti-inflammatory and antioxidative properties [34, 35]. Niacin protects lung function impairment through the activation of SIRT1 and reduced pulmonary oxidative stress and lung inflammation [36]. A study indicated that niacin, vitamins A and D, and dietary fiber associated with on average better FEV1 in chronic smokers [37]. A high dose of niacin attenuated lung inflammation, suppressed proinflammatory cytokine release, reduced histologic lung damage, and improved survival [38]. While the exact molecular mechanism of the association between dietary niacin intake and the risk of COPD requires further investigation.

This study possesses several strengths. Firstly, it was a large-scale investigation examining the association between dietary niacin intake and the prevalence of COPD in the US middle-aged and older individuals. Secondly, stringent screening of measurement methods and protocols was conducted within the NHANES database. Thirdly, adjustments for potential confounders were made, ensuring robust and stable conclusions across different subgroups. Additionally, to assess the robustness of the results, we performed several sensitivity analyses.

Nevertheless, there are certain limitations to our research. Firstly, the NHANES population consists of Americans and may not be representative of other populations. Secondly, due to the cross-sectional study design of NHANES, establishing a causal relationship between dietary niacin intake and COPD is not feasible. Thirdly, the assessment of 24-hour dietary intake may not precisely reflect an individual participant’s lifetime diet. However, 24-hour dietary recall is widely acknowledged as the most suitable method for estimating the average intake of a population [39]. Additionally, we mitigated potential errors by averaging two 24-hour diet recall interviews. Fourthly, the diagnosis of COPD relies on self-reporting, introducing the possibility of recall bias.

## Conclusion

This study established a reverse association between dietary niacin intake and COPD in the American population middle-aged and older individuals. Our results suggest that niacin may be a significant nutrient influencing COPD. Further prospective studies are necessary to validate and corroborate our findings.

## Supporting information

S1 Table Baseline characteristics of study population. (DOCX)

S2 Table Association between dietary niacin intake and COPD in participants without extreme energy intake(n = 7040). (DOCX)

S3 Table Association between dietary niacin intake and COPD after excluding extremes of niacin intake Mean±3SD (n = 7057). (DOCX)

S4 Table Association between dietary niacin intake and COPD after multiple imputation for missing covariates (n = 8767). (DOCX)

S5 Table Weighted logistics regression of the association between dietary niacin intake and COPD. (DOCX)
