# Supplementary material for: Association between dietary niacin intake and chronic obstructive pulmonary disease among American middle-aged and older individuals: A cross-section study
Source: PLoS One. 2024 Nov 21;19(11):e0312838. doi: 10.1371/journal.pone.0312838 (PMC11581289; doi:10.1371/journal.pone.0312838)
Supplement: S1 Table — (DOCX) [file pone.0312838.s001.docx]

**Table S1. Baseline characteristics of study population**

| **Variables** | **Total (n = 7170)** | **Non-COPD (n = 6336)** | **COPD (n = 834)** | ***p*** |
| --- | --- | --- | --- | --- |
| Sex, n (%) |  |  |  | 0.005 |
| Male | 3407 (47.5) | 3049 (48.1) | 358 (42.9) |  |
| Female | 3763 (52.5) | 3287 (51.9) | 476 (57.1) |  |
| Age, years, Mean ± SD | 59.5 ± 11.9 | 59.1 ± 11.9 | 62.9 ± 11.5 | <0.001 |
| Physical activity, n (%) |  |  |  | <0.001 |
| Sedentary | 3900 (54.4) | 3321 (52.4) | 579 (69.4) |  |
| Moderate | 2048 (28.6) | 1855 (29.3) | 193 (23.1) |  |
| Vigorous | 1222 (17.0) | 1160 (18.3) | 62 (7.4) |  |
| Smoking status, n (%) |  |  |  | <0.001 |
| Never | 3817 (53.2) | 3588 (56.6) | 229 (27.5) |  |
| Former | 2145 (29.9) | 1819 (28.7) | 326 (39.1) |  |
| Current | 1208 (16.8) | 929 (14.7) | 279 (33.5) |  |
| Race/ethnicity, n (%) |  |  |  | <0.001 |
| Mexican American | 926 (12.9) | 875 (13.8) | 51 (6.1) |  |
| Non-Hispanic white | 3106 (43.3) | 2598 (41) | 508 (60.9) |  |
| Non-Hispanic black | 1501 (20.9) | 1358 (21.4) | 143 (17.1) |  |
| Others | 1637 (22.8) | 1505 (23.8) | 132 (15.8) |  |
| Education level, n (%) |  |  |  | <0.001 |
| Less than high school | 1402 (19.6) | 1205 (19) | 197 (23.6) |  |
| High school | 1670 (23.3) | 1431 (22.6) | 239 (28.7) |  |
| More than high school | 4098 (57.2) | 3700 (58.4) | 398 (47.7) |  |
| Marital status, n (%) |  |  |  | <0.001 |
| Married | 4211 (58.7) | 3824 (60.4) | 387 (46.4) |  |
| living with a partner | 331 (4.6) | 292 (4.6) | 39 (4.7) |  |
| Living alone | 2628 (36.7) | 2220 (35) | 408 (48.9) |  |
| Family income, n (%) |  |  |  | <0.001 |
| Low（PIR<1.3） | 1970 (27.5) | 1618 (25.5) | 352 (42.2) |  |
| Medium（1.3≤PIR<3.5） | 2802 (39.1) | 2472 (39) | 330 (39.6) |  |
| High（PIR≥3.5） | 2398 (33.4) | 2246 (35.4) | 152 (18.2) |  |
| Body mass index, n (%) |  |  |  | <0.001 |
| <25 kg/m2 | 1694 (23.6) | 1514 (23.9) | 180 (21.6) |  |
| 25 to <30 kg/m2 | 2432 (33.9) | 2213 (34.9) | 219 (26.3) |  |
| ≥30 kg/m2 | 3044 (42.5) | 2609 (41.2) | 435 (52.2) |  |
| Total energy, Kcal, Mean ± SD | 2028.3 ± 921.1 | 2036.4 ± 918.1 | 1967.2 ± 941.8 | 0.041 |
| Hypertension, n (%) | 3465 (48.3) | 2962 (46.7) | 503 (60.3) | <0.001 |
| High cholesterol, n (%) | 3391 (47.3) | 2922 (46.1) | 469 (56.2) | <0.001 |
| Diabetes, n (%) | 1470 (20.5) | 1226 (19.3) | 244 (29.3) | <0.001 |
| Coronary heart disease, n (%) | 466 (6.5) | 321 (5.1) | 145 (17.4) | <0.001 |
| Stroke, n (%) | 389 (5.4) | 303 (4.8) | 86 (10.3) | <0.001 |
| Cancer, n (%) | 1013 (14.1) | 832 (13.1) | 181 (21.7) | <0.001 |
| Niacin intake (mg/d) | 23.8 ± 11.6 | 24.0 ± 11.7 | 22.2 ± 11.2 | <0.001 |
| Serum cotinine, ng/mL, Median (IQR) | 0.0 (0.0, 0.5) | 0.0 (0.0, 0.2) | 0.1 (0.0, 209.5) | <0.001 |
