# Supplementary material for: Association between dietary niacin intake and chronic obstructive pulmonary disease among American middle-aged and older individuals: A cross-section study
Source: PLoS One. 2024 Nov 21;19(11):e0312838. doi: 10.1371/journal.pone.0312838 (PMC11581289; doi:10.1371/journal.pone.0312838)
Supplement: S2 Table — (DOCX) [file pone.0312838.s002.docx]

**Table.S2 Association between dietary niacin intake and COPD in participants without extreme energy intake(n=7040)**

| Variable | **Crude model** | |  | **Model 1** | |  | **Model 2** | |  | **Model 3** | |
| --- | --- | --- | --- | --- | --- | --- | --- | --- | --- | --- | --- |
|  | **OR (95%CI)** | ***p*** |  | **OR (95%CI)** | ***p*** |  | **OR (95%CI)** | ***p*** |  | **OR (95%CI)** | ***p*** |
| Quartile |  |  |  |  |  |  |  |  |  |  |  |
| Q1 (≤16.14) | 1(Ref) |  |  | 1(Ref) |  |  | 1(Ref) |  |  | 1(Ref) |  |
| Q2(16.15-21.83) | 0.86 (0.71~1.05) | 0.135 |  | 0.86(0.71~1.05) | 0.143 |  | 0.97 (0.78~1.2) | 0.784 |  | 0.96(0.77~1.19) | 0.714 |
| Q3(21.84-28.82) | 0.72 (0.59~0.88) | 0.001 |  | 0.73 (0.59~0.9) | 0.004 |  | 0.78 (0.62~0.99) | 0.037 |  | 0.77 (0.61~0.98) | 0.033 |
| Q4 (≥28.83) | 0.63 (0.51~0.78) | <0.001 |  | 0.71 (0.57~0.9) | 0.004 |  | 0.76 (0.58~1) | 0.05 |  | 0.75 (0.57~0.99) | 0.043 |
| *P* for trend |  | <0.001 |  |  | 0.001 |  |  | 0.017 |  |  | 0.015 |

**Abbreviations:** COPD, chronic obstructive pulmonary disease; Q, quartiles; OR, odds ratio; CI, confidence interval; Ref: reference.

The crude model was not adjusted for covariates.

Model I was adjusted for sex, age, race/ethnicity.

Model2 was adjusted for sex, age, race/ethnicity, family income, physical activity, smoking status, education level, marital status, body mass index, serum cotinine, total energy.

Model 3 was adjusted for sex, age, race/ethnicity, family income, physical activity, smoking status, education level, marital status, body mass index, serum cotinine, total energy, hypertension, high cholesterol, diabetes, coronary heart disease, stroke, cancer.
