# Supplementary material for: Association between dietary niacin intake and chronic obstructive pulmonary disease among American middle-aged and older individuals: A cross-section study
Source: PLoS One. 2024 Nov 21;19(11):e0312838. doi: 10.1371/journal.pone.0312838 (PMC11581289; doi:10.1371/journal.pone.0312838)
Supplement: S3 Table — (DOCX) [file pone.0312838.s003.docx]

**Table.S3. Association between dietary niacin intake and COPD after excluding extremes of niacin intake Mean±3SD (n=7057)**

| Variable | Crude model | |  | Model 1 | |  | Model 2 | |  | Model 3 | |
| --- | --- | --- | --- | --- | --- | --- | --- | --- | --- | --- | --- |
|  | OR (95%CI) | p-Value |  | OR (95%CI) | p-Value |  | OR (95%CI) | p-Value |  | OR (95%CI) | *p* |
| Quartile |  |  |  |  |  |  |  |  |  |  |  |
| Q1 (≤16.0) | 1(Ref) |  |  | 1(Ref) |  |  | 1(Ref) |  |  | 1(Ref) |  |
| Q2(16.01-21.69) | 0.82 (0.67~1) | 0.046 |  | 0.81 (0.67~0.99) | 0.044 |  | 0.93 (0.75~1.15) | 0.513 |  | 0.93 (0.75~1.15) | 0.504 |
| Q3(21.70-28.53) | 0.74 (0.61~0.9) | 0.003 |  | 0.75 (0.61~0.92) | 0.007 |  | 0.82 (0.65~1.03) | 0.088 |  | 0.82 (0.65~1.03) | 0.09 |
| Q4 (≥28.54) | 0.62 (0.51~0.77) | <0.001 |  | 0.7 (0.56~0.87) | 0.002 |  | 0.75 (0.57~0.99) | 0.041 |  | 0.74 (0.56~0.98) | 0.036 |
| *P* for trend |  | <0.001 |  |  | 0.001 |  |  | 0.025 |  |  | 0.023 |

**Abbreviations:** COPD, chronic obstructive pulmonary disease; Q, quartiles; OR, odds ratio; CI, confidence interval; Ref: reference.

The crude model was not adjusted for covariates.

Model I was adjusted for sex, age, race/ethnicity.

Model2 was adjusted for sex, age, race/ethnicity, family income, physical activity, smoking status, education level, marital status, body mass index, serum cotinine, total energy.

Model 3 was adjusted for sex, age, race/ethnicity, family income, physical activity, smoking status, education level, marital status, body mass index, Serum cotinine, total energy, hypertension, high cholesterol, diabetes, coronary heart disease, stroke, cancer.
