# Supplementary material for: Association between dietary niacin intake and chronic obstructive pulmonary disease among American middle-aged and older individuals: A cross-section study
Source: PLoS One. 2024 Nov 21;19(11):e0312838. doi: 10.1371/journal.pone.0312838 (PMC11581289; doi:10.1371/journal.pone.0312838)
Supplement: S4 Table — (DOCX) [file pone.0312838.s004.docx]

**Table.S4.****Association between dietary niacin intake and COPD after multiple imputation for missing covariates（n=8767）**

| **Variable** | **Crude model** | |  | **Model 1** | |  | **Model 2** | |  | **Model 3** | |
| --- | --- | --- | --- | --- | --- | --- | --- | --- | --- | --- | --- |
|  | **OR (95%CI)** | ***p*-Value** |  | **OR (95%CI)** | ***p*-Value** |  | **OR (95%CI)** | ***p*-Value** |  | **OR (95%CI)** | ***p*** |
| Quartile |  |  |  |  |  |  |  |  |  |  |  |
| Q1 (≤15.98) | 1(Ref) |  |  | 1(Ref) |  |  | 1(Ref) |  |  | 1(Ref) |  |
| Q2(15.99-21.73) | 0.79 (0.66~0.94) | 0.009 |  | 0.79 (0.66~0.94) | 0.01 |  | 0.88 (0.73~1.07) | 0.21 |  | 0.88 (0.73~1.07) | 0.213 |
| Q3(21.74-28.86) | 0.73 (0.61~0.88) | 0.001 |  | 0.74 (0.61~0.89) | 0.001 |  | 0.81 (0.66~0.99) | 0.042 |  | 0.81 (0.66~1) | 0.049 |
| Q4 (≥28.87) | 0.63 (0.52~0.76) | <0.001 |  | 0.7 (0.57~0.85) | 0.001 |  | 0.76 (0.59~0.97) | 0.025 |  | 0.76 (0.59~0.98) | 0.033 |

**Abbreviations:** COPD, chronic obstructive pulmonary disease; Q, quartiles; OR, odds ratio; CI, confidence interval; Ref: reference.

The crude model was not adjusted for covariates.

Model I was adjusted for sex, age, race/ethnicity.

Model2 was adjusted for sex, age, race/ethnicity, family income, physical activity, smoking status, education level, marital status, body mass index, serum cotinine, total energy.

Model 3 was adjusted for sex, age, race/ethnicity, family income, physical activity, smoking status, education level, marital status, body mass index, Serum cotinine, total energy, hypertension, high cholesterol, diabetes, coronary heart disease, stroke, cancer.
