# Supplementary material for: Association between dietary niacin intake and chronic obstructive pulmonary disease among American middle-aged and older individuals: A cross-section study
Source: PLoS One. 2024 Nov 21;19(11):e0312838. doi: 10.1371/journal.pone.0312838 (PMC11581289; doi:10.1371/journal.pone.0312838)
Supplement: S5 Table — (DOCX) [file pone.0312838.s005.docx]

**Table.S5.Weighted logistics regression of the association between dietary niacin intake and COPD**

| Variable | Crude model | |  | Model 1 | |  | Model 2 | |  | Model 3 | |
| --- | --- | --- | --- | --- | --- | --- | --- | --- | --- | --- | --- |
|  | OR (95%CI) | p-Value |  | OR (95%CI) | p-Value |  | OR (95%CI) | p-Value |  | OR (95%CI) | *p* |
| Quartile |  |  |  |  |  |  |  |  |  |  |  |
| Q1 (≤15.98) | 1(Ref) |  |  | 1(Ref) |  |  | 1(Ref) |  |  | 1(Ref) |  |
| Q2(15.99-21.73) | 0.98 (0.70~1.37) | 0.881 |  | 1.02 (0.72~1.45) | 0.915 |  | 1.19 (0.85~1.67) | 0.30 |  | 1.20 (0.87~1.65) | 0.256 |
| Q3(21.74-28.86) | 0.61 (0.42~0.89) | 0.011 |  | 0.66 (0.45~0.95) | 0.027 |  | 0.71 (0.49~1.03) | 0.068 |  | 0.74 (0.50~1.07) | 0.106 |
| Q4 (≥28.87) | 0.56 (0.38~0.83) | 0.005 |  | 0.69 (0.45~1.06) | 0.086 |  | 0.79 (0.54~1.15) | 0.202 |  | 0.79 (0.53~1.16) | 0.21 |
| P for trend |  | <0.001 |  |  | 0.017 |  |  | 0.023 |  |  | 0.039 |

**Abbreviations:** COPD, chronic obstructive pulmonary disease; Q, quartiles; OR, odds ratio; CI, confidence interval; Ref: reference.

The crude model was not adjusted for covariates.

Model I was adjusted for sex, age, race/ethnicity.

Model2 was adjusted for sex, age, race/ethnicity, family income, physical activity, smoking status, education level, marital status, body mass index, serum cotinine, total energy.

Model 3 was adjusted for sex, age, race/ethnicity, family income, physical activity, smoking status, education level, marital status, body mass index, Serum cotinine, total energy, hypertension, high cholesterol, diabetes, coronary heart disease, stroke, cancer.
